# The Mechanical Properties and Water-Reducing and Retarding Mechanism of a Xylonic Cement Admixture

**DOI:** 10.3390/ma16227096

**Published:** 2023-11-09

**Authors:** Feng Han, Kaijian Huang, Yang Wei, Jian Han, Yong Xu

**Affiliations:** 1 College of Civil Engineering, Nanjing Forestry University, Nanjing 210037, China; hanfeng2678@sina.com (F.H.); wy78@njfu.edu.cn (Y.W.); 2 Jiangsu Co-Innovation Center of Efficient Processing and Utilization of Forest Resources, College of Chemical Engineering, Nanjing Forestry University, Nanjing 210037, China; nl_hanjian@njfu.edu.cn (J.H.); xuyong@njfu.edu.cn (Y.X.)

**Keywords:** xylonic acid, water reducer, slow setting mechanism, water reducing mechanism

## Abstract

This study explores the mechanical properties, as well as the water-reducing and setting delay mechanism, of a novel xylonic acid-based water reducer applied to cementitious materials. Four xylonic acid water reducers were synthesized in this study: XACa (PX) from pure xylose, XACa (HS) from hemicellulose hydrolysate, XANa (PX) from pure xylose, and XANa (HS) from hemicellulose hydrolysate. These were generated through the whole-cell catalysis of Gluconobacter oxydans bacteria, using pure xylose and hemicellulose hydrolysate as substrates. The findings indicate that the xylonic acid-based water reducer can attain a water-reducing capability between 14% and 16% when the dosage (expressed as a mass fraction of cement) is roughly 0.2%. In initial and final setting tests, XACa (PX) demonstrated a pronounced retarding influence at admixture levels below 0.15%, reaching its apex at 0.10%. This delayed the initial setting time by 76% and the final setting time by 136% relative to the control group. However, a slight pro-setting effect was noted beyond a 0.2% dosage. In the compressive and flexural tests of concrete, under the same slump, the XA group improved its mechanical properties by 5% to 10% compared to the SodiuM lignosulfonate (SL) group. In the air content and chloride ion migration resistance tests, the XA group reduced the air content by 38% compared to the SL group, but also increased the data of rapid chloride migration (D_RCM_) by 16%. Characterization studies revealed that the carboxyl and hydroxyl groups in xylonic acid undergo chemisorption with the Si-O bonds on the surface of cement particles. These groups interact with the Si-O bonds on cement particles, contributing to water-reducing effects and delaying the setting process by impeding Ca^2+^ ion aggregation in the calcium-silicate-hydrate gel. Its significant water-reducing effect, adjustable setting time, and excellent mechanical and durability properties suggest its viability as an alternative to lignosulfonate series water-reducing agents.

## 1. Introduction

The use of concrete admixtures is one of the most effective and widely adopted strategies for enhancing the workability of concrete [1,2]. At present, the widely used concrete water reducing agents on the market are mostly naphthalene-based water reducing agents, polycarboxylic acid water reducing agents, and simple combinations of the two with other admixtures such as early strength agents, with a small portion being lignosulfonate-based water reducing agents. The dispersion mechanism of a polycarboxylate water-reducing agent on cement is a dual mechanism of electrostatic repulsion and steric hindrance [3]. Although the effect is outstanding, its cost is high, and there is no unified standard for its compound formula, which has mixed quality. With respect to the naphthalene-based water-reducing agents, hydrophilic groups dissociated from them are directionally adsorbed on the surface of cement particles [4], making it easy to associate with water molecules in the form of hydrogen bonds to form water, prevent direct contact between cement particles, and provide lubrication between particles. Although naphthalene-based water-reducing agents are relatively cheap, with the rise of large-scale construction such as highway bridges, mechanical mixing and pumping of concrete have exposed a fatal drawback: rapid loss of slumps. Both production processes will pollute the environment to varying degrees. Environmentally friendly lignosulfonate, as an anionic surfactant, has good adsorption and dispersion properties. When added to cement slurry, it can dissociate into macromolecular anions and metal cations. Anions with negative charges adsorb on the surface of positively charged cement particles [5,6,7,8,9], which can generate a certain electrostatic repulsion and disperse the cement particles. As “low-carbon development” has gradually become a global consensus for economic development, which has led to heightened demands for environmentally friendly additives in terms of raw materials and production processes.

Currently, the predominant biomass water reducer in use is lignosulfonate. Derived as a by-product of the sulfite process in wood pulp papermaking, lignosulfonate carries the advantages of being environmentally friendly and cost-effective. Nevertheless, it is not without its shortcomings, such as a low water-reducing efficiency and a high level of air entrainment and retardation, factors that constrain its broader application.

In addition, Maimaiti [10] treated waste cellulose to obtain microcrystalline cellulose (MCC), then with BS to obtain SBC, which was experimentally proved to be a retarded water reducer. As the degree of polymerization (DP) of MCC rises, the degree of substitution of SBC and concrete fluidity decrease, but the fluidity of concrete is not negatively correlated with the DP of MCC. Only when the DP of MCC < 96 and the degree of substitution > 0.375, SBC has the water-reducing ability, and the water-reducing and dispersing effect is best when DP = 45. The retardation effect may be due to the fact that the BS in SBC did not react completely with the -OH in cellulose, which made these groups form complexes with the inorganic particles in cement to cover the surface of the cement particles, and it is also possible that the -OH in cellulose is bonded with the water molecules to form hydrogen bonds, which formed a solventized water film on the surface of the cement particles and prevented the hydration reaction from proceeding. Rouzi [11] prepared a cellulose-based water reducer (CS) in a dichloromethane solution using cellulose cotton paddle meal as the base material and chlorosulfonic acid as the sulfonating agent. When the tension of the ratio of sulfonating agent and cellulose is 0.6, the water reducer obtained at room temperature for 3 h has the best performance. The surface CS aqueous solution decreases with an increase in the concentration of CS water reducer, and it has a certain air-entraining effect on the concrete. CS water reducer is adsorbed on the surface of cement particles, which makes the surface of cement particles have the same charge and then produces a repulsive effect resulting in the dispersion of cement particles, resulting in a water-reducing and dispersing effect. All of the above biomass water reducers have the problems of low water-reducing efficiency (no more than 10%) and difficulty going into production. Finding more efficient and environmentally friendly water-reducing agents is still necessary.

In the current research on xylonic acid-based (XA) water-reducing agents, Zhou’s [12] experiment roughly explored the water-reducing efficiency and setting time of XA. Compared with Zhou’s experimental results on water-reducing efficiency and air content after the addition of XA water-reducing agent, the relevant data are roughly the same as in this article. However, in terms of setting time, Zhou obtained experimental results that the setting time increases to 15 h with the increase of the addition amount between 0 and 0.40%. In the initial stage of this article, the initial and final setting times of cement paste are different from the results of Zhou, which makes it necessary to conduct more accurate and systematic experiments on the XA water-reducing agent to verify its performance.

Lignocellulose, a biomass resource with an approximate global annual yield of 180 billion tons [13], chiefly comprises cellulose, hemicellulose, and lignin. It possesses benefits such as carbon neutrality, economic viability, and broad availability. In order to fully exploit the potential of straw-based biomass resources, it is imperative to transform cellulose and hemicellulose into their corresponding monosaccharides for microbial conversion into resource-derived products [12,14,15,16,17,18]. This study employed sulfuric acid treatment as a means to eliminate lignin, thus liberating more cellulose and hemicellulose, disrupting the straw structure, augmenting the contact surface between microorganisms or enzymes and straw, and enhancing the conversion efficiency of straw-like biomass.

In this investigation, the studied xylonic acid (XA) water reducers were synthesized using pure xylose (PX) and xylose-rich hemicellulose hydrolysate (HS) as substrates. XA bears a chemical structure akin to gluconate, a well-known water-reducing and retarding agent in the cement and concrete industries. Consequently, XA, with its hydroxyl and carboxyl groups, offers potential as a novel concrete admixture. Characteristically, the xylonic acid water reducer is non-toxic, non-hazardous, eco-friendly, and biodegradable throughout its entire production process, inclusive of both raw materials and catalysts. When contrasted with lignosulfonate-based biomass water reducers, the xylonic acid water reducer presents remarkable benefits such as low gassing, minimal admixture requirement, high efficiency, control over setting time, and extensive application prospects [19,20,21].

This manuscript offers a succinct overview of the production methodology of xylonic acid water reducers and probes their water-reducing efficiency and optimal admixture quantity through experimental investigation. The outcomes hint at the potential substitutability of sodium lignosulfonate (SL) water reducers with these new developments.

## 2. Experiment

### 2.1. Experimental Materials

The cement is P.O 42.5 Hailuo brand ordinary silicate cement, which is produced by Nanjing Longtan Town Cement Factory; its specific gravity is 3.1. The fine aggregate is medium sand with a fineness modulus of 2.8, and the coarse aggregate is basalt with a grain size of 15–25 mm.

In the compressive strength test of mortar, which is to select the better reducers, W/C is 0.284. In the compressive and flexural strength tests of concrete, the ratio of cement, fine aggregate, and coarse aggregate in the concrete mixture is 1:1.5:2.55. Maintaining uniformity of slumps by varying the dose of water-reducing agent and the amount of water used. The water absorption of coarse aggregate is 1.24%.

Four distinct xylonic acid water reducers, specifically XACa (PX), XACa (HS), XANa (PX), and XANa (HS), were generated via whole-cell fermentation of the strain Gluconobacter oxydans after neutralization with NaOH and Ca(OH)_2_ to reach a pH conducive to the strain’s survival. The hemicellulose hydrolysate was derived from the treatment of agricultural residues such as corn stover and corn cobs with sulfuric acid. Gluconobacter oxydans, a bacterium of significant industrial relevance, is known for its production of aldehydic acids like xylogluconic acid (XA), which are utilized in this investigation. The pH of XA water reducers is 7~8. The specific gravity of XA water reducers is 0.35.

Water-reducing agents are listed in Table 1:

The basic properties and mineral composition of the cement are shown in Table 2 and Table 3. The particle size distribution of aggregates is listed in Table 4 and Table 5.

### 2.2. Methods

#### 2.2.1. Determination of Water Consumption and Setting Time for Standard Consistency of Cement Paste

The determination of the setting time for the cement paste adhered to the GB 1346-2001 (Chinese Standard) [22], The initial setting standard was defined when the probe of the Automatic Vicatometer descended by (34 ± 2) mm, and the final setting was marked when the probe penetrated the cement surface by (3 ± 1) mm.

#### 2.2.2. Characterization of Cement Paste Samples

The cement paste with the same standard consistency was prepared and cured for 24 h in a constant temperature and humidity curing oven. A small portion was taken out and immersed in ethanol for an additional 24 h to halt the hydration reaction. Subsequently, it was transferred to an oven for drying. The surface appearance of the cement was observed using an environmental scanning electron microscope (Environmental Scanning Electron Microscope, Quanta 200, FEI, Hillsborough, OR, USA). Another portion was pulverized, milled to a 200-mesh consistency, and similarly placed in ethanol to stop the hydration process for 24 h. Subsequently, the XRD patterns of cement hydrates were measured using a multifunctional X-ray diffractometer (XRD Ultima IV, Rigaku, Tokyo, Japan)after an additional 24 h of suspension in ethanol.

#### 2.2.3. Compressive Strength Test of Mortar

The experiment was conducted adhering to the guidelines of JGJ/I 70-2009 (Chinese Standard) [23]. The samples were prepared to a size of 70.7 mm × 70.7 mm × 70.7 mm for the test, with a lateral compression test being executed (Pressure Testing Machine, HTC-1068, Shenzhen Wance Test Equipment Co., Shenzhen, China) following a maintenance period of 7 days.

#### 2.2.4. Compressive and Flexural Strength Test of Concrete

The experiment was conducted adhering to the guidelines of GB 50204-2015 (Chinese Standard) [24]. The samples were prepared to sizes of 100 mm × 100 mm × 100 mm for the compressive test, and 100 mm × 100 mm × 400 mm for the flexural test, with the lateral test being executed (Pressure Testing Machine, HTC-1068, Shenzhen Wance Test Equipment Co., Shenzhen, China) following a maintenance period of 7 and 28 days.

#### 2.2.5. Concrete Air Content Test

In alignment with GB 50203-2011 (Chinese Standard) [25], a concrete air content meter(Direct Reading Concrete Air Content Tester, LC-615A, Jianyanhuace (Hangzhou) Technology Co., Hangzhou, China) was utilized. The concrete mix was evenly loaded into the measurement bowl and vibrated for 15–30 s until it became compact. The surface was then scraped to be flat, ensuring no air bubbles were present. The bowl was covered and filled with water until it reached the outlet, at which point the tap and exhaust valve were closed. The bowl was pressurized with air until the gauge pressure exceeded 0.1 MPa, precisely regulated with a trimmer valve. After actuating the valve lever 1–2 times, the pressure gauge reading was recorded. Utilizing the calibration curve, the value of air content A_1_ was ascertained, and the aggregate air content C was determined using the water pressure method. The final air content A was calculated as A = A_1_ − C.

#### 2.2.6. Concrete Resistance to Chloride Ion Permeability Test

The examination was conducted in conformity with GB/T 50082-2009 (Chinese Standard) [26], utilizing the Rapid Chloride Migration Coefficient Method (RCM)(Concrete Chloride Ion Diffusion Coefficient Tester, CABR-RCM, Jianyan Building Materials Co., Beijing, China). A cylindrical specimen with a diameter of (100 ± 1) mm and a height *L* = (50 ± 2) mm was initially placed in a Ca(OH)_2_ vacuum saturator for 24 h. Subsequently, it was enclosed in a rubber sleeve and introduced into the RCM experimental apparatus. Each sleeve was injected with 300 mL of NaOH solution with a concentration of 0.3 mol/L, while the cathodic experimental tank was filled with 12 L of NaCl solution with a concentration of 10%. Upon connecting the power supply’s positive and negative terminals and energizing for *t* = 24 h, the energizing voltage *U* and temperature *T* were recorded. The specimen was dissected along the diameter of the circular surface, followed by the application of an AgNO_3_ solution. The average value of chloride ion penetration depth *Xd* was noted, and the non-stationary chloride ion migration coefficient *D_RCM_* of the concrete was calculated using the prescribed Equation (1).

(1) $$D_{RCM}=\frac{0.239*(273+T)L}{(U-2)t}(X_{d}-0.238\sqrt{\frac{(273+T)LX_{d}}{U-2}})$$

## 3. Results and Discussion

### 3.1. Influence of Different Xylonic Acid Water Reducer on Water Consumption

**Figure 1:**
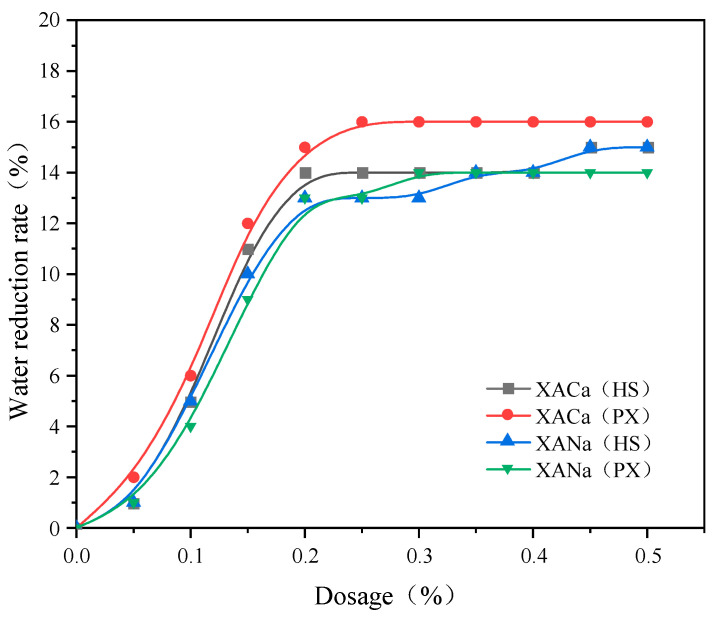
Effect of XA dosage on the water reduction rate.

XACa (PX), XACa (HS), XANa (PX), and XANa (HS) were prepared using pure xylose and hydrolysate as reaction substrates, with calcium carbonate and sodium hydroxide as neutralizing substances, respectively. The water-reducing effects were evaluated based on the water consumption required to reach standard consistency, and the results are presented in Figure 1.

From Figure 1, it is evident that the water-reducing capacity of xylonic acid water reducer gradually increases with higher dosages until reaching a saturation point. When the dosage is below 0.2%, the water consumption of cement to achieve standard consistency decreases with an increasing dosage of xylonic acid water reducer. However, at dosages ranging from 0.2% to 0.3%, the rate of increase in water-reducing capacity slows down. Beyond 0.3% dosage, all four water reducers exhibit maximum water-reducing effects. The water-reducing effects of the four xylonic acid water reducers are comparable, with XACa (HS), obtained by neutralizing Ca(OH)_2_ with hydrolysate as the substrate, exhibiting the highest water-reducing effect. However, considering the effective content determination of the powder formulation and test errors, the effects of the four xylonic acid water reducers were considered equivalent for the time-setting.

### 3.2. Impact of Xylonic Acid (HS) Water Reducer Dosage on the Setting Time of Cement Paste

**Figure 2:**
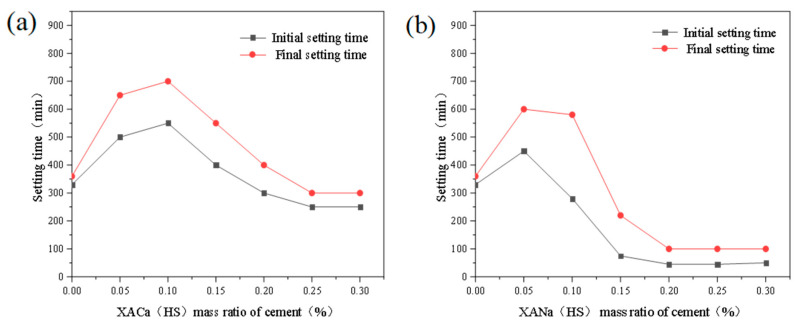
Effect of XA dosage on setting time: (**a**) XACa (HS); (**b**) XANa (HS).

The initial and final setting times of cement paste were investigated by introducing xylonic acid water reducers at dosage levels ranging from 0% to 0.30% in increments of 0.05%. The tests were conducted using standard consistency water consumption (water-cement ratio of 0.284), 99% humidity in the curing chamber, and 20 °C. The results are presented in Figure 2.

From Figure 2, it can be observed that both XACa (PX) and XANa (PX) exhibit a retarding effect on the cement paste at a dosage of 0.05%. The retarding effect of XACa (PX) reaches its peak at 0.10%, increasing the initial setting time by approximately 76%. Conversely, the retarding effect of XANa (PX) shows a decreasing trend, and at 0.15%, it demonstrates a pro-setting effect, reducing the initial setting time by around 79%. With increasing dosage, the retarding effect of XANa (PX) remains consistent, while the retarding effect of XACa (PX) gradually diminishes and ceases after reaching 0.20%.

### 3.3. Impact of Xylonic Acid (PX) Water Reducer on the Setting Time of Cement Paste

**Figure 3:**
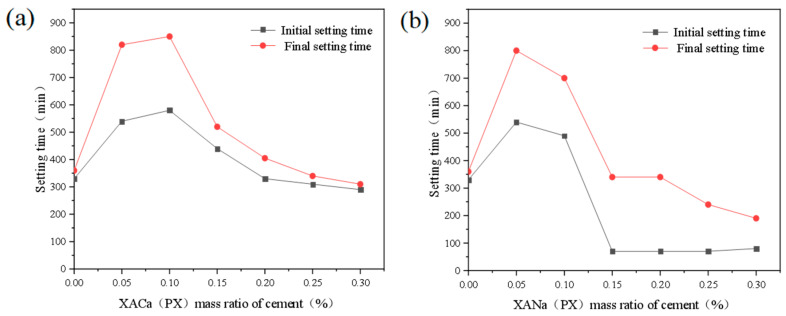
Effect of XA dosage on setting time: (**a**) XACa (PX); (**b**) XANa (PX).

Similarly, the effects of XACa (PX) and XANa (PX) on the final setting time of cement slurry are shown in Figure 3.

Align with their initial setting effects. At a dosage of 0.05%, both water reducers extend the final setting time by approximately 128%. At a dosage of 0.10%, XACa (PX) exhibits a slightly improved retarding effect, while the retarding effect of XANa (PX) starts to decrease. With further dosage increases, XANa (PX) demonstrates a setting-promoting ability at 0.15%, followed by XACa (PX) at 0.25%. Additionally, XANa (PX) exhibits coagulation-promoting ability at 0.25%. Notably, XANa (PX) displays a stronger pro-setting ability, reducing the final setting time of cement paste by 47% at 0.30%. Zhou [12] obtained experimental results that the addition amount is between 0% and 0.40%, and the final setting time extends to 15 h with the increase of the addition amount. This may be due to the different purity of xylic acid compared to this article.

When the dosage of xylonic acid is sufficiently high, it promotes the generation of a large amount of Ettringaite (referred to as AFt), consumes a significant amount of mixing water, rapidly thickens the slurry, and, together with the bridging effect of AFt, accelerates cement setting. Conversely, with lower dosages of xylonic acid, the generation of AFt is limited, resulting in delayed formation of tricalcium silicate (C_3_S) hydration and calcium silicate hydrate (CSH) gel, thus slowing down the cement setting process. This hypothesis was verified in subsequent characterization experiments.

The test results of xylonic acid obtained using hydrolysate as the substrate raw material demonstrate similar effects, where low dosages retard coagulation and high dosages promote coagulation. However, the coagulation time of both hydrolysate groups is slightly shorter than that of the pure xylose group. This can be attributed to trace impurities present in the hydrolysate, including but not limited to acids (formic acid, acetic acid, levulinic acid, butyric acid, and 4-hydroxybenzoic acid) and aldehydes (5-hydroxymethyl furfural, furfural, vanillin, butyraldehyde, and 5-hydroxybenzaldehyde), which hinder the reaction between xylonic acid and cement mineral components, thereby partially inhibiting the promoting effect of xylonic acid on C_3_A hydrolysis.

**Figure 4:**
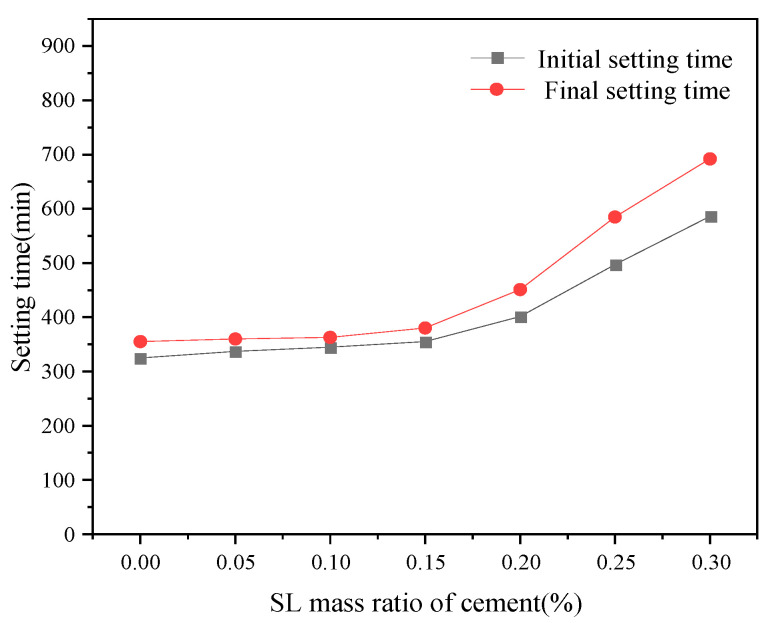
Effect of SL dosage on setting time.

Comparing the retarding effect of xylonic acid with lignosulfonate water reducers with certain retarding effects (Figure 4), it was found that the retarding effect is similar, but the dosing stage of the performance is different. With the increase in SL dosing, the retarding effect also increases.

SL has been utilized as a retarding agent for oil well cement for decades. Although the exact mechanism by which lignosulfonate delays silicate formation is not yet clear, it is speculated that this mechanism is a combination of adsorption and nucleation [27]. According to studies, sulfonates and hydroxyl groups can be adsorbed on the CSH gel layer of hydrated cement. This means the sulfonates and hydroxyl groups existing in the lignosulfonate can be mixed into the CSH gel layer, resulting in changes in the morphology of the CSH gel and a more impermeable structure. This will result in a waterproof effect, slowing down further hydration. The more sulfonates and hydroxyl groups it provides, the tardier the hydration proceeds, which led to the proportional linear result in the figure above.

### 3.4. Effect of XA Dosage on the Compressive Strength of Mortar

**Figure 5:**
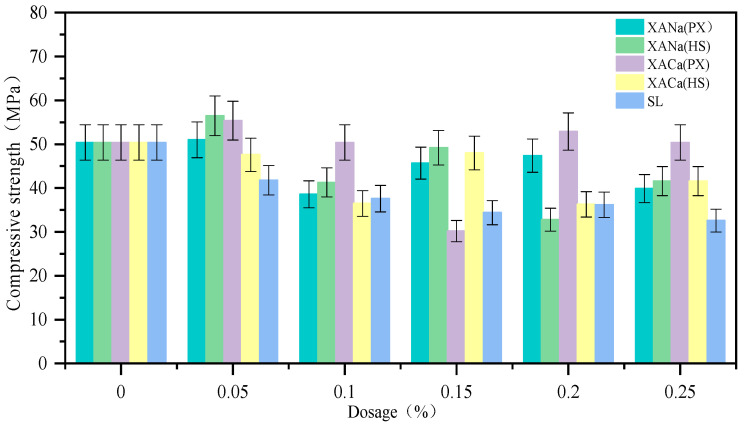
Effect of XA dosage on compressive strength.

The influence of four xylonic acid water reducers on the compressive strength of test blocks with mortar exhibited varied performance, as depicted in Figure 5. At a low admixture of 0.05%, the compressive strength improved; however, it decreased to some extent as the admixture increased. At the optimal admixture of 0.2%, the hydrolysate group demonstrated superior performance with minimal strength reduction. Notably, XACa (HS) even exhibited a slight increase in compressive strength. This can be attributed to the retardation effect of xylonic acid at low to medium doping levels, which slows down the hydration rate and leads to lower 7-day strength formation. Considering the results of the aforementioned experiments, it is evident that the HS group outperformed the others. Therefore, subsequent experiments in this study employed two hydrolysates as xylonic acid water reducers.

In the case of the SL group, the compressive strength of mortar consistently decreased with increasing dosage. At its optimal admixture of 0.2~0.3%, the 7-day compressive strength decreased by approximately 30%. This decrease can be attributed to the air entrainment side effect of SL, which inversely impacts the compressive strength of mortar as air content rises.

### 3.5. Effect of Different Water Reducers on the Compressive and Flexural Properties of Concrete

**Figure 6:**
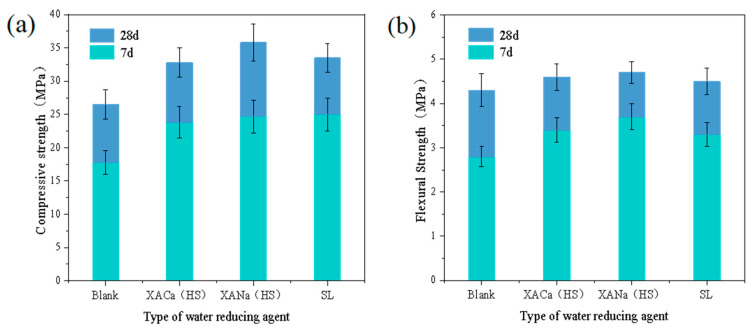
Effect of different reducers on the compressive and flexural strengths: (**a**) compressive strength; (**b**) flexural strength.

Concrete compressive and flexural tests were conducted at 7 days and 28 days, using the water-reducing capacity of each water reducer obtained from the previous cement paste experiments (XA for 0.2% and SL for 0.3%), while maintaining a concrete mix slump of 100 mm. Analysis of the data in Figure 6 reveals significant improvements in the mechanical properties of concrete with XANa (HS) and XACa (HS) at the same slump level. Compared to the blank group, the xylonic acid water reducer demonstrated enhancements of approximately 35% and 27% in the 7-day and 28-day compressive strengths of concrete, respectively. Moreover, the flexural strength of concrete exhibited improvements of approximately 25% and 8% at 7 days and 28 days, respectively. Comparatively, the overall improvement over the SL water reducer ranged from 5% to 10%. In terms of compressive strength, the results are not compared with Zhou’s results due to different concrete mix ratios.

The improvement of compressive strength and flexural strength of concrete by three types of water-reducing agents is an inevitable result of the reduction of the water-cement ratio, and the enhancement of compressive strength is more obvious than the enhancement of flexural strength. This is because there are many cracks in the normal microscopic level of the concrete, and this kind of crack is unavoidable and randomly distributed [28]. The damage to concrete is due to the development of cracks, which tend to close instead of developing under compression, while cracks develop under tension, leading to concrete damage. This effect can be significantly improved by adding reinforcement.

### 3.6. Effect of Various Water Reducers on Air Content and Resistance to Chloride Ion Penetration in Concrete

The entrainment of air in concrete serves as a vital parameter that receives differential evaluation across distinct application contexts. On one side, the deliberate inclusion of gas can subtly augment the flexural strength of concrete and notably enhance its durability. This process, which introduces a myriad of minute air bubbles uniformly distributed at an optimal gas content and suitably spaced, effectively elongates the migration route of chloride ions, thereby establishing a blockade against chloride ion penetration. As a result, the concrete’s resistance to chloride ion infiltration is fortified [29,30]. Furthermore, its resilience against freeze–thaw cycles also sees significant improvement. However, on the flip side, the presence of excessive entrapped air can precipitously diminish the concrete’s strength and potentially instigate engineering mishaps.

**Figure 7:**
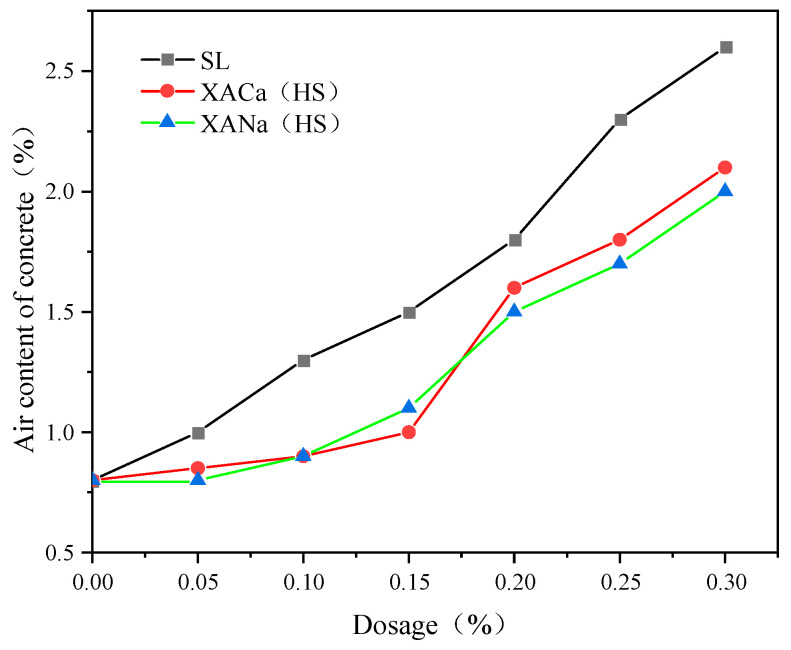
Effect of reducer type and dosage on the air content of concrete.

As illustrated in the accompanying Figure 7, the xylonic acid water reducer consistently introduces less air into the concrete compared to the SL group as the admixture level increases. This reduction in air content subsequently leads to a decrease in the resistance to chloride ion penetration during the chloride ion penetration resistance experiments [29,31]. The results are basically consistent with Zhou.

In the experiment assessing the resistance to chloride ion penetration (Table 6), the rapid chloride migration coefficient method (RCM) was employed. The results indicated that the migration coefficient of xylonic acid, dosed at 0.2%, was significantly lower than that of the control group and slightly lower than that of the SL group. Thus, the xylonic acid water reducer can be considered a viable alternative to SL as a water reducer in environments where a water reducer with a lower chloride ion content is required.

### 3.7. Microscopic Characterization

**Figure 8:**
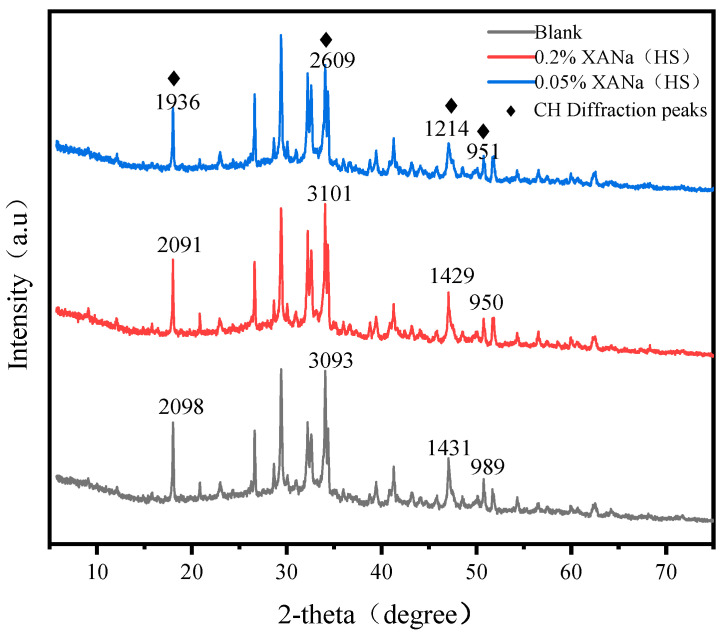
XRD of three dosage XANa(HS).

The intensity of the characteristic peak of calcium hydroxide (CH) in the control group demonstrated similar levels to the 0.2% XANa (HS)-doped samples, while it was significantly higher than that observed in the 0.05% XANa (HS) samples, as indicated by the XRD plots (Figure 8).

**Figure 9:**
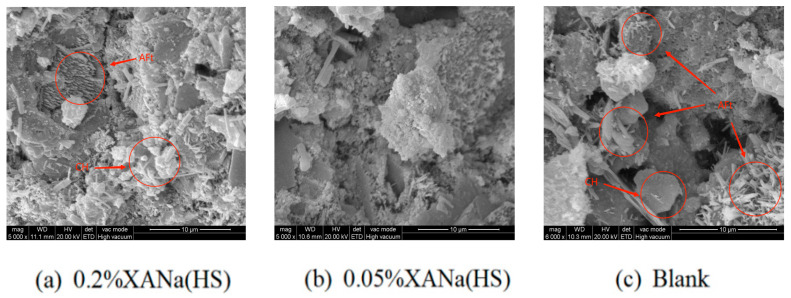
SEM of three dosage XANa(HS): (**a**) 0.2%; (**b**) 0.05%; (**c**) blank.

Electron micrographs further confirmed these findings (Figure 9), revealing increased formation of AFt and CH in the 1-d cement paste with 0.2% XANa (HS) compared to the control group. Conversely, the 1-d cement paste with 0.05% XANa (HS) exhibited considerably less AFt, and no CH formation was observed.

Based on the micro-level characterization conducted through SEM and XRD analyses, it was determined that the addition of a higher dosage of xylonic acid water reducer accelerated the formation of AFt and CH during the 1-d cement hydration process in comparison to the control group. Conversely, the addition of a lower dosage of xylonic acid water reducer slowed down AFt formation and hindered the formation of CH [32,33]. This effect was attributed to the inhibitory role of the lower xylonic acid dosage on the hydration of C_3_S and its binding with Ca^2+^ ions, preventing the formation of CH as a crystalline nucleation agent. In contrast, the higher xylonic acid dosage promoted the initial hydration process of C_3_A, accelerating AFt formation and enabling the rapid development of a certain strength within three hours. Additionally, it facilitated the formation of an AFt-phase film on the surface of cement particles. As the AFt-phase film thickened and generated crystallization pressure, it eventually ruptured, leading to the subsequent formation of a new AFt phase in the damaged region. This cyclic process resulted in a denser structure. The early end of the induction period accelerates the formation of CH. Consequently, the cement paste with 0.2% xylonic acid water reducer exhibited higher compressive strength compared to the control group, despite having the same water-cement ratio. Conversely, the lower dosage of xylonic acid delayed AFt formation and interacted with Ca^2+^ ions in the CSH gel [34], preventing their aggregation and retarding CSH growth, thereby requiring more than 600 min to achieve initial setting.

**Figure 10:**
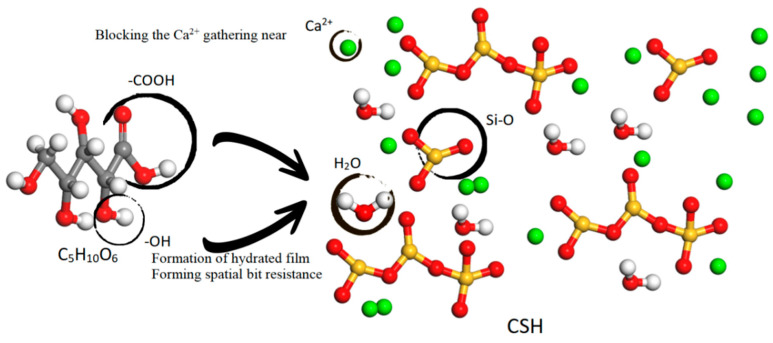
Schematic diagram of the water reduction and retarding mechanism.

According to Figure 10 (based on the Pellenq model [35]), carboxyl and hydroxyl groups exhibit a propensity to chemically adsorb to the Si-O bonds present on the surface of cement particles [34,36]. Additionally, hydrophilic groups combine with water molecules [33,34,35], resulting in cement particles and water sharing similar groups. This interaction between identical groups provides a lubricating effect among cement particles, while the hydrophilic end of the xylonic acid molecule interacts with water, reducing surface tension [37,38,39]. Consequently, water molecules diffuse and disperse more effectively in the concrete mixture, improving the fluidity of the cement slurry and acting as a water-reducing agent.

## 4. Conclusions

In the present investigation, four variants of xylonic acid-based water-reducing agents—XACa (PX), XACa (HS), XANa (PX), and XANa (HS)—were synthesized leveraging whole-cell catalysis of Gluconobacter oxydans, employing pure xylose and hemicellulose hydrolysate as substrates. The main conclusions obtained can be summarized as follows:

(1) Findings demonstrated that these xylonic acid water-reducing agents delivered a water reduction efficiency in the range of 14% to 16% when the dosage approximated 0.2%. Experiments determining the initial and final setting times of cement paste demonstrated that XA manifested a significant retarding influence at doses below 0.15%, peaking at 0.10%. It extended the initial setting time by 76% and the final setting time by 136% relative to the control group. However, at dosages of 0.2% and beyond, it demonstrated a minor acceleration effect.
(2) In tests assessing the mechanical properties of mortar, the XA(HS) group exhibited superior compressive strength compared to the XA(PX) group at equivalent dosages. Moreover, the XA(HS) group exhibited commendable performance in compressive and flexural, air-entrainment, and chloride ion penetration resistance experiments, showcasing performance akin to SL.
(3) The adsorption of the carboxyl and hydroxyl groups in xylonic acid onto the Si-O bonds on the cement particle surface escalated the fluidity of the cement paste. Moreover, the chemisorption of hydroxyl and carboxyl groups with the Si-O bonds on the cement particle surface provided water-reducing functionality, thereby influencing the formation of CSH and delaying the setting process.
(4) The XA(HS) group demonstrated significant water-reducing effects, the capacity to regulate setting time, and satisfactory outcomes in mechanical durability tests, thus indicating its potential to supplant lignosulfonate-based water-reducing agents.

Given that the experiments conducted in this article are very limited, more application experiments are needed in the future to verify the practical application feasibility of XA water-reducing agent, such as the dry shrinkage of concrete after addition and its compatibility with steel bars or steel fiber composite materials.

## Figures and Tables

**Table 1:** The water reducers.

Particulars	XACa (PX)	XANa (PX)	XACa (HS)	XANa (HS)	SL
Category	Xlonic acid				Sodium lignosulfonate
Molecular formula	C_5_H_10_O_6_				C_20_H_24_Na_2_O_10_S_2_
Reaction materials	Pure xylose		Hemicellulose hydrolysate		Lignin + Na_2_SO_3_
Reaction environment	Ca(OH)_2_	NaOH	Ca(OH)_2_	NaOH	NaOH
Recommended dose	0~0.2%				0~0.3%
Efficiency of water reduction	0~16%				0~11%
Other functions	Promoting/retarding hydration, air-entraining				Retarding hydration, air-entraining

**Table 2:** Chemical composition and content of cement.

Component	LOI	Al_2_O_3_	CaO	MgO	Fe_2_O_3_	SO_2_	SO_3_
Content (%)	2.98	5.85	70.45	1.24	2.97	19.00	2.83

**Table 3:** Physical and mechanical properties of cement.

Water Consumption of Standard Consistency (%)	Setting Time (min)		Compressive Strength (MPa)		Flexural Strength (MPa)	
	Initial	Final	3 d	28 d	3 d	28 d
28.6%	328	362	27.8	54.2	5.4	8.6

**Table 4:** Particle size distribution of coarse aggregates.

Material Specifications	Proportion (%)	Percentage Passing (%)							
		31.5	26.5	19	16	13.2	9.5	4.75	2.36
20~30 mm	45	98	67.4	3.9	0.8	-	-	-	-
10~20 mm	55	100	100	96.9	75.0	6.9	5.3	0.6	-

**Table 5:** Particle size distribution of fine aggregates.

Material Specifications	Fineness Modulus	Percentage Passing (%)					
		2.0	1.6	1.0	0.5	0.16	0.08
Medium sand	2.8	100	93	67	23	13	1

**Table 6:** Effect of different additives on the air content and *D_RCM_* of concrete.

Additive Dosing (%)	Slumps (mm)	W/C	Air Content (%)	*D_RCM_* (×10^−12^ m^2^/s^−1^)
Blank	100	0.60	0.8	20.18
0.1%XACa(HS)	100	0.57	1.2	18.02
0.2%XACa(HS)	100	0.52	1.6	14.71
0.1%XANa(HS)	100	0.57	1.2	17.98
0.2%XANa(HS)	100	0.52	1.6	14.57
0.15%SL	100	0.57	1.5	17.33
0.3%SL	100	0.54	2.6	12.38

## Data Availability

Data are contained within the article.
